# Pregnancies after vaginal radical trachelectomy (RT) in patients with early invasive uterine cervical cancer: results from a single institute

**DOI:** 10.1186/s12884-020-02949-1

**Published:** 2020-04-25

**Authors:** Shota Shinkai, Shinichi Ishioka, Tasuku Mariya, Yuya Fujibe, Miseon Kim, Masayuki Someya, Tsuyoshi Saito

**Affiliations:** Department of Obstetrics and Gynecology, Sapporo Medical University, S1, W16, Chuou-ku, Sapporo, Hokkaido 0608543 Japan

**Keywords:** Cervical cancer, Fertility, Pregnancy, Trachelectomy, Pregnancy, Vaginal radical trachelectomy, Uterine cervix

## Abstract

**Background:**

Radical tracheletomy (RT) with pelvic lymphadenectomy has become an option for young patients with early invasive uterine cervical cancer who desire to maintain their fertility. However, this operative method entails a high risk for the following pregnancy due to its radicality.

**Methods:**

We have performed vaginal RT for 71 patients and have experienced 28 pregnancies in 21 patients. They were followed up carefully according to the follow-up methods we reported previously. Their pregnancy courses and prognoses after the pregnancy were retrospectively reviewed.

**Results:**

All the vaginal RTs were performed safely without serious complications, including 6 patients who underwent the operation during pregnancy. The median time to be pregnant after RT was 29.5 months. 13 patients (46%) became pregnant without artificial insemination by husband or assisted reproductive technology. Cesarean section was performed for all of them. The median time of pregnancy was 34 weeks, and emergent cesarean section was performed for 7 pregnancies (25%). The median birth weight was 2156 g. Four patients had trouble with cervical cerclage, and they suffered from sudden premature preterm rupture of the membrane (pPROM) during the second trimester of pregnancy. We underwent transabdominal cerclage (TAC) for all of them and careful management for the prevention of uterine infection was performed. One patient had a recurrence of cancer during pregnancy.

**Conclusions:**

Both the obstetrical prognosis and oncological prognosis after vaginal RT have become favorable for pregnant patients after vaginal RT.

## Background

Radical trachelectomy (RT) with pelvic lymphadenectomy, originally reported by Dargent et al. in 1988 [1], has become a new treatment option for young patients in Japan with early invasive uterine cervical cancer who desire preservation of their fertility [2–4]. This operative modality is recently performed even during pregnancy [5]. Recent data indicate that the oncologic results following RT are comparable to the results following standard radical hysterectomy (RH) [6–8].

Almost 15 years has passed since we started vaginal RT in our institute, and we have performed 71 vaginal RTs so far. Among those who underwent this operation, there have been 28 pregnancies in 21 patients. However, as was reported in various studies, with pregnancy after RT there still remains a high risk of preterm labor and subsequent preterm birth. This is because of the lack of mechanical support of the residual cervix and ascending infection and chorioamnionitis (CAM) caused by disruption of the endocervical glands and reduced secretion of mucus after the radical operation [9, 10]. Although the optimal obstetrical management of these patients remains controversial, the follow-up modality for pregnant patients after vaginal RT in our department has been improved year by year. However, they still usually need long-term obstetrical treatments over the course of the pregnancy.

We believe it is important to examine this technique not only from the oncological viewpoint but also from the obstetrical one to improve both the oncological and the obstetrical prognoses of patients with this disease. In this report, we summarize the clinical profile of pregnancy after vaginal RT in our institute and review the obstetrical management of pregnancy after vaginal RT. This is the first cumulative study of pregnancy after vaginal RT in Japan.

## Methods

Twenty-eight pregnancies in 21 patients after vaginal RT with pelvic lymphadenectomy in our institute between 2007 and 2018 are reviewed in this study. The study protocol was approved by the review board and the ethics committee in our institute (approval number 05–26). A written informed consent was also obtained from each patient.

The clinical characteristics of the patients are summarized in Table 1 and the clinical indications for vaginal RT in our hospital are summarized in Table 2. All of the patients except one had International Federation of Gynecology and Obstetrics (FIGO) 2009 stage 1A2-1B1 uterine cervical cancer. Clinical stage 1B2 and higher stages are beyond the indication for RT in our hospital, although RT was performed for a patient with stage IIA who strongly desired the preservation of fertility after repeated explanations to her and her family. Adjuvant chemotherapy using paclitaxel and carboplatin was also used for her during pregnancy and after cesarean section. Assessment of pelvic lymph node metastasis was performed by laparotomy or laparoscopy at the time of vaginal RT. For pregnant patients, it was performed by magnetic resonance imaging (MRI) before the operation and the lymph node sampling at the time of the operation.

**Table 1:** Clinical characteristics and evolution of pregnancies in patients who had a pregnancy at the time or after RT

	Case	Age^a^	Pregnancy after RT (months).	Stage	Pregnancy^b^	Histology^c^	Admission (weeks)	Delivery (weeks)	Causes of termination^d^	CAM^e^	Baby weight (g)	Special remarks^f^
1^st^trimesterabortion	7–2	30s	82	1B1	IVF	SCC	06	6	sp.abortion	n.a	n.a	
	19–2	30s	111	1B1	IVF	adeno	n.a	9	sp.abortion	n.a	n.a	
2nd trimester abortion	3–1	30s	64	1B1	IVF	SCC	15	19	pPROM	+	n.a	
	7–1	30s	29	1B1	IVF	SCC	15	18	pPROM	+	n.a	
	19–1	30s	51	1B1	IVF	adeno	10	19	pPROM	+	n.a	
2nd trimester birth	1–2	30s	62	1B1	IUI	ad.-sq	14	26	pPROM	+	878	
	2–1	30s	6	1B1	IVF	SCC	20	23	pPROM	+	588	
	2–2	40s	6	1B1	IVF	SCC	14	24	pPROM	+	846	TAC
	12	30s	n.a	1B1	natural	SCC	15	26	recurrence	―	882	In preg.
3rd trimester birth	1–1	30s	8	1B1	natural	ad.-sq	17	32	pPROM	+	1991	
	3–2	30s	30	1B1	IVF	SCC	14	34	liver dys.	―	1862	TAC
	4	20s	21	1B1	clomifene	SCC	14	35	scheduled	―	2138	
	5	30s	21	1B1	natural	SCC	14	34	scheduled	+	2294	
	6	30s	24	1A2	natural	ad.-sq	19	34	scheduled	―	2520	
	7–3	40s	99	1B1	IVF	SCC	14	33	scheduled	―	2720	TAC
	8	30s	n.a	1B1	natural	SCC	15	34	scheduled	―	2112	In preg.
	9	40s	55	1B1	IVF	SCC	17	35	scheduled	―	2126	
	10	30s	10	1B1	natural	SCC	15	35	scheduled	―	2470	
	11	30s	19	1B1	IUI	adeno	15	34	scheduled	―	2326	
	13	30s	15	1B1	natural	SCC	16	35	scheduled	―	2052	
	14	30s	20	1B1	IVF	SCC	14	30	scheduled	―	1224/1449	Twin
	15	30s	n.a	1B1	natural	adeno	20	35	scheduled	―	2156	In preg.
	16	30s	n.a	1B1	natural	adeno	26	34	scheduled	―	2328	In preg.
	17	40s	n.a	1B1	natural	SCC	16	35	scheduled	―	2400	In preg.
	18	20s	n.a	IIA	natural	SCC	17	30	scheduled	―	1188	Chemo.
	19–3	30s	123	1B1	IVF	adeno	14	33	scheduled	―	1912	TAC
	20	30s	31	1B1	natural	SCC	16	35	scheduled	―	2588	
	21	30s	29	1A2	IVF	SCC	15	31	pPROM	+	1684	

^a^as to age, 20s means 20–29 y.o, 30s,eams 30–39 y.o, and 40s means 40–49 y.o

^b^as to pregnancy, “IUI” means intra uterine insemination by husband, “IVF” means in vitro fertilization, “clomifene” means oral administration of clomifene, and “natural” means pregnancy without such infertility treatments

^c^as to histology, “SCC” means squamous cell carcinoma, “adeno” means adenocarcinoma, and “ad.-sq.” means adenosquamous carcinoma.”sp.abortion” means spontaneous abortion

^d^as to causes of termination, “pPROM” means premature preterm rupture of membrane, “scheduled” means scheduled cesarean section, “liver dys” means liver dysfunction

^e^“CAM” means chorioamnionitis

^f^“TAC” means transabdominal cerclage. TAC was performed for patients with “TAC” after their second trimester loss of pregnancy. “chemotherapy”, means administration of anticancer drugs, paclitaxel and carboplatin. “In preg.” means RT during pregnancy. “Twin” means twin pregnancy. “Chemo” means chemotherapy for the patient during pregnancy

**Table 2:** Preoperative indication of vaginal RT

1. Patients≦45 years with the strong desire to preserve fertility.	
2.International Federation of Gynaecologists and Obstetricians (FIGO) stage 1A1 with vascular space involvement, stage 1A2 or stage 1B1	
2. Lesion size≦2 cm	
3. Squamous histology or adenocarcinoma (including adenosquamous carcinoma).	
4. No involvement of the upper endocervical canal as determined by colposcopy or magnetic resonance imaging (MRI), and no evidence of lymph node metastasis.	

Vaginal RT itself was performed based on a modification of the procedure of Dargent et al. Amputation height was preoperatively determined by transvaginal ultrasonography and careful search of lesion area by colposcopy for each patient.

The procedure started with lymphadenectomy by laparotomy or laparoscopy, and then vaginal RT was performed. Briefly, a rim of vaginal mucosa was delineated circumferentially and excised so that the anterior and posterior mucosae could cover the cervix. The vesicovaginal space was defined laterally on each side. Then the descending branches of the uterine arteries and the cardinal ligaments were cut at the level of Type II hysterectomy. After this procedure, we usually amputated the uterine cervix 10 mm below the internal os of the uterus. A nylon suture was placed around the residual cervix to support cervical strength, and Strumdorf sutures were placed to cover the surface of the cervix. Follow-up of the cancer was performed by periodical checking of cytology, tumor markers, and CT scans. Tumor marker examination and CT scans were not usually performed for pregnant patients.

Vaginal RT for pregnant patients was also conducted according to the same procedure. However, as the patients were pregnant, we confirmed the fetal status by ultrasonography soon after the operation, and careful check of the amount of the bleeding from the vaginal wound and the existence of leakage of amniotic fluid were performed. Additionally, prophylactic continuous intravenous administration of tocolytic agents was performed at least for 1 week after RT.

We performed transabdominal cerclage (TAC) for four patients who experienced second trimester fetal losses due to troubles with the transvaginal cerclage placed at the time of vaginal RT. Details of the operative procedure are described in our previous report [11].

As for the management of pregnant patients after vaginal RT, there are no international guidelines so far. Prevention of cervical and uterine infection, and prevention of preterm uterine contraction are a key to avoid pPROM and the following preterm birth. We recommend that patients enter hospitalization early in the second trimester of the pregnancy even if there are no signs of threatened abortion. Daily vaginal disinfection with 0.3% chlorhexidine, the administration of an ulinastatin suppository, and weekly examination of vaginal flora, the granulocyte elastase level and oncofetal fibronectin in vaginal secretion, the measurement of uterine cervical length by transvaginal ultrasonography, and weekly intramuscular administration of a hydroxyprogesterone caproate depot were performed for the patients under moderate bed rest. Serum CRP and general blood examinations were also done weekly. In cases with a Nugent score > 5, and those that were cervical elastase or fibronectin positive, transvaginal administration of metronidazole was performed. Tocolysis started when the patients felt abdominal pain/tension, or the uterine cervical length shortened to < 2 cm.

Ritodrine hydrochloride and/or magnesium sulfate were intravenously administered as tocolytic agents. Nifedipine was also orally administered. Considering the maternal physical and psychological conditions from the long-term bed rest and tocolysis, and to avoid the sudden occurrence of premature preterm rupture of membrane (pPROM), cesarean section was usually scheduled at 34–35 weeks of pregnancy. For one patient who had recurrence during pregnancy, standard total abdominal RH was performed at the time of cesarean section.

**Fig. 1:**
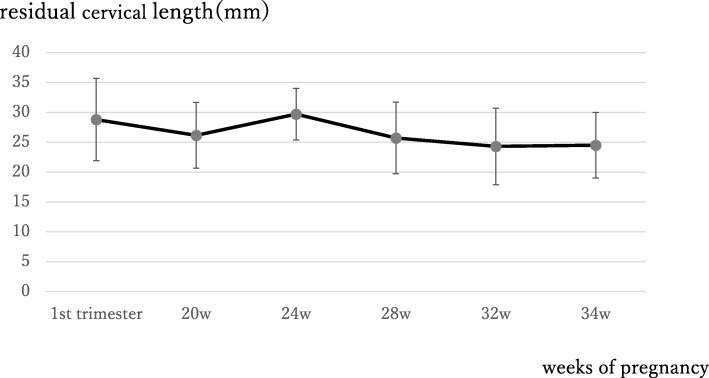
Changes in the cervical length during pregnancy in patients after vaginal RT. Cervical length at each point is expressed as “average ± SE” for all the patients measured in this study

Measurement of uterine cervical length was performed weekly for each patient from 14 weeks of pregnancy until the end of pregnancy. Changes of the cervical length during pregnancy were expressed as “average ± SE” using Microsoft EXCEL 2016 in Fig. 1.

## Results

We performed 71 vaginal RTs for patients with early invasive uterine cervical cancer from 2006 to 2018 at Sapporo Medical University. Six of the operations were performed during the period from 16 to 26 weeks of pregnancy (Nos. 8, 12, 15, 16, 17, and 18). Specimens of all the pregnant patients had cancer free margin > 20 mm.

The clinical characteristics of the patients are summarized in Table 1. In total, we experienced 28 deliveries by 21 patients, with 24 live births, including a case of twin pregnancy (patient 14). The overall pregnancy rate among the patients was 29.6%. The median time to each pregnancy after RT was 29.5 months (range 6–123 months). Thirteen pregnancies (46.4%) did not require artificial insemination or assisted reproductive technology. The median time of pregnancy, excluding first trimester abortion, was 34 weeks (range 18–35 weeks).

### Oncological results

The patient’s median age was 32 years (range 23–46 years). Eighteen had stage 1B1 tumors and 2 had stage 1A2 tumors. For patient 18, who had a stage 2A tumor, vaginal RT was performed due to her strong desire for conservative treatment. Histologically, 15 patients had squamous cell carcinoma, 4 had adenocarcinoma, and 2 had adenosquamous carcinoma. All the patients are alive without any signs of recurrence at present. One patient (No. 12) who underwent RT during pregnancy had recurrence at 24 weeks of pregnancy. She underwent cesarean section and RH at 26 weeks of pregnancy, and she is alive without further recurrence.

### Obstetric results

#### Summary

Cesarean section was performed safely for each patient. For the incision to the uterus, a transverse incision (not a vertical one) was made to the lower part of the uterine body. All the fetuses, except for those that ended in first trimester abortion, had normal fetal growth over the course of pregnancy. No adverse effects on fetal growth of RT were detected in any patient. We performed TAC for four patients (Nos. 2, 3, 7, and 19) who had troubles with the cerclage, and we could perform scheduled cesarean section for three of them. One patient (No. 2) gave preterm birth at 24 weeks of pregnancy due to pPROM in spite of TAC.

#### First trimester fetal losses

First trimester fetal losses due to spontaneous abortion occurred in 2 patients. One patient (No. 19) was treated with curettage after slight dilatation of the remaining uterine cervix. However, for another patient (No. 7), fetal components were not excreted for almost 2 months after the diagnosis of abortion, and the elevation of blood HCG was detected. She was administered methotrexate (MTX) and the fetal components were excreted naturally thereafter.

#### Second trimester losses

Three pregnancies ended in second trimester losses. At the time of pregnancy, they had various troubles of the transvaginal cerclage that was placed at the time of vaginal RT. Patients 3 and 7 became pregnant long after vaginal RT. Patient 3 had a slack cerclage due to the degradation of the thread, and the thread was lost for patient 7 at the time of pregnancy. Patient 19, who underwent vaginal RT at another hospital, did not receive a transvaginal cerclage. These patients suffered from sudden pPROM without any signs of vaginal infection at 18–19 weeks of pregnancy. Markers for genital tract inflammation such as the granulocyte elastase level and the oncofetal fibronectin level in the vaginal secretions were mostly within the normal range over the pregnancy. BV scores were also mostly normal before the occurrence of pPROM. Cesarean section was performed for them. Pathologically, chorioamnionitis (CAM) was detected in each of them.

#### Emergent cesarean section in the late second to early third trimester of pregnancy

Emergent cesarean section in the late second trimester to the third trimester of pregnancy was performed for 7 pregnancies in 5 patients. The reason for the emergent cesarean section was pPROM for 5 pregnancies in 3 patients (patient 1, 2, and 21). Especially, patient 1 and patient 2 suffered from pPROM twice. For the second pregnancy of patient 3, liver dysfunction, probably due to the long-term administration of ritodrine, was the cause of the emergent cesarean section. One patient who underwent vaginal RT during pregnancy (patient 12) had recurrence in the residual cervix 8 weeks after RT, at 24 weeks of pregnancy. She underwent cesarean section with radical hysterectomy (RH) at 26 weeks of pregnancy, as described above. The children of patients 1, 3, 12 and 21 show normal growth so far. Especially the child of patient 12 was cared at neonatal intensive care unit (NICU) for longer than 3 months, and he discharged without any serious complications. The first child of patient 2 has cerebral palsy, and the second one died 3 days after birth due to respiratory disorders. CAM was detected for all the patients with pPROM.

#### Scheduled cesarean section

Scheduled cesarean section was performed for 16 pregnancies in 16 patients. The median time of pregnancy was 34 weeks (range 30–35 weeks). The median body weight of the newborns was 2156 g (range 1188–2720 g). Most patients continued their pregnancies until 34 weeks or longer. Control of preterm labor was successful with bed rest and the administration of ritodrine, magnesium sulfate, and/or nifedipine. Patient 14 underwent cesarean section at 30 weeks of pregnancy due to preterm labor with a twin pregnancy, and patient 18 underwent it at 30 weeks of pregnancy due to the necessity for adjuvant chemotherapy. The mothers’ postoperative courses were uncomplicated. None of them have had recurrence after giving birth.

### Obstetrical management

#### Uterine cervical length and infectious signs

Changes of uterine cervical length measured by transvaginal ultrasonography over the pregnancy are presented in Fig. 1. Cervical length tended to become shorter with the progression of the pregnancy. However, it was maintained at 2 cm or more over the pregnancy in most cases.

#### Tocolysis

We performed long-term tocolysis for the patients who showed symptoms of preterm labor (Table 3). Twenty-one patients (75%) used ritodrine-based intravenous treatments.

**Table 3:** Tocolytic treatments during pregnancy

	First trimester losses	Second trimester losses	Emergent CS	Scheduled CS	Total
div ritodrine^a^	0	3	5	9	17
div ritodrine+ Mg^b^	0	0	1	2	3
div ritodrine+ nifedipine	0	0	0	1	1
oral ritodrine^a^	0	0	1	4	5
no drugs	2	0	0	0	2

^a^div ritodrine means intravenous drip of ritodrine. Oral ritodrine means oral administration of ritodrine

^b^Mg means magnesium sulfate

## Discussion

This study demonstrated both the safety and the difficulties of the obstetrical management of pregnant patients after vaginal RT in our institute. For the approach to the uterine cervix, abdominal RT is performed more frequently than vaginal RT in Japan. Many of the reports thus far in Japan have been those using the abdominal approach [3, 12]. This is the first cumulative report regarding pregnancies after vaginal RT in Japan.

Of the 21 patients, who underwent vaginal RT during pregnancy, one (4.8%) had a recurrence at 24 weeks of gestation and underwent cesarean section with radical hysterectomy. She is currently free of disease. No other patient has had recurrence to date after pregnancy. Our recurrence rate in pregnant women corresponded to the reports of 4.8% after vaginal RT by Plante et al. [9], and 3.7% after abdominal RT by Li et al. [13]. As we reported recently, expansion of vaginal RT even for pregnant patients is a practical option now [5]. We believe that operative procedures of vaginal RT for pregnant patients to remove the uterine cervix are more conservative and simpler than abdominal RT for them because vaginal RT makes it possible to cut the uterine cervix and the parametrium without laparotomy. In addition, the pregnancy rate without artificial insemination or assisted reproductive technology was very high in our study. The conservative approach to the uterus of vaginal RT seems to have made possible this better pregnancy rate.

Most patients who became pregnant after vaginal RT in this study could continue their pregnancy until the time of scheduled cesarean section. As is well known, pregnancy after RT has higher risks of preterm birth. Although there are no international guidelines for the following-up of pregnant patients after RT, the basics of the maternal management for patients after RT are bed rest and the prevention of uterine cervical infection. The necessity of bed rest under hospitalization and its effectiveness are, however, still controversial [14]. Bed rest for a long time is not only a burden for patients but can also be a cause of deep vein thrombosis (DVT). However, we still believe that bed rest is essential to prevent emergencies in patients after RT.

Prevention of infection is another key to improve the obstetrical prognosis.

It is said that 25–60% of preterm births are attributable to maternal infections, and bacterial vaginosis (BV) has been suggested to be a risk factor for preterm birth [15, 16]. BV is a condition in which the normal, lactobacillus-predominant vaginal flora is replaced with anaerobic bacteria such as Gardonerella vaginalis and Mycoplasma hominis [17]. BV has also been reported to be associated with pPROM and CAM [18]. Therefore, the prevention and the treatment of BV during pregnancy might have an impact to improve obstetrical prognosis in patients after RT. We as well as Speiser et al. administered Metronidazole vaginal tablets for the treatment of BV during pregnancy [19]. Ito et al. reported the usefulness of oral probiotics to reduce the occurrence of BV and the following pPROM for a patient after RT [20].

We usually cut the uterine cervix about 10 mm below the internal os of the uterus. Such a large cut of the uterine cervix causes disruption of the endocervical glands, reduces secretion of mucus, and promotes cascades from BV to CAM. Furthermore, it can be a cause of reduced mechanical support of the residual cervix. Therefore, the transvaginal cerclage of the residual cervix, which is usually placed at the time of radical RT, plays an important role in maintaining pregnancy [10]. Troubles with this cerclage can become a cause of second trimester fetal losses and early preterm birth. Patients with a slack cerclage usually suffered from sudden pPROM without elevation of infectious markers such as BV, cervical elastase, and fibronectin. Shepherd et al. also reported the same types of pPROM in patients after RT [21]. How does it occur? The uterine cavity has been believed to be in a sterile condition. However, recent analysis using next-generation sequencing of the 16 s ribosomal RNA gene revealed the existence of various bacteria there [22]. Kyono et al. reported that a considerable percentage of the non-Lactobacillus-dominated microbiota, which is impossible to detect by routine bacterial culture, affects the occurrence of infertility and preterm abortion [23]. We believe such subclinical intrauterine infections, which are undetectable by routine bacterial culture examination, might exist in patients with a slack cerclage after RT.

As for the treatment of patients with preterm uterine contraction, there are differences between Japan and other western countries. We still use long term administration (> 72 h) of ritodrine hydrochloride and/or magnesium sulfate for them, but most western countries do not undergo long term tocolysis for them. Tocolytic agents are usually used for at most 72 h. Instead, progesterone gels or pessaries will be used for preterm uterine contraction as described in a review by Tirlapur et al. [24]. Ritodrine hydrochloride has not been used in western countries except Japan due to its complications anymore. However we believe, if it is used carefully and with lower doses (50-200 μg/min), it might be effective to improve obstetrical prognosis of patients after RT without serious complications.

What can gynecologic oncologists do to reduce such obstetrical troubles? Transabdominal uterine cervical cerclage (TAC), originally proposed by Benson and Durfee in 1965, actually seems to improve the obstetrical prognosis for patients who have troubles with the cerclage [11].

Large amputation of the uterine cervix can be a cause of preterm birth. Therefore, more conservative and less invasive operations for young patients are in great demand all over the world. However, there are only a few reports on less invasive operations for patients with early invasive uterine cervical cancer so far. Schmeler et al. reported that parametrium invasion occurred in less than 1% of patients with 1A2-1B1 with a < 2 cm tumor without lymphovascular invasion [25]. And our study revealed that a simple trachelectomy 20 mm not 10 mm below the internal os with pelvic lymphadenectomy might be possible for stage 1A2 patients [26]. These results might indicate the possibility of more conservative trachelectomy such as simple trachelectomy with a shorter amputation of the uterine cervix which lead to the improvement of the obstetrical prognosis for stage 1A2 patients [26, 27].

The cooperation of gynecologic oncologists and obstetricians thus has a great impact on improving the obstetrical prognosis for patients after RT. In addition, it is important to consolidate these high risk pregnant patients in large medical facilities that can provide comprehensive care.

## Conclusions

We have performed 71 vaginal RTs for patients with early invasive uterine cervical cancer, and have experienced 28 pregnancies in 21 patients so far. Both the obstetrical prognosis and oncological prognosis after vaginal RT have become favorable for pregnant patients after vaginal RT under the appropriate management.

## Data Availability

Data will be available from corresponding author upon resendable request.

## Abbreviations

RT: Radical trachelectomy
pPROM: Premature preterm rupture of the membrane
TAC: Transabdominal cerclage
RH: Radical hysterectomy
CAM: Chorioamnionitis
FIGO: Federation of Gynecology and Obstetrics
MRI: Magnetic resonance imaging
MTX: Methotrexate
NICU: Neonatal intensive care unit
DVT: Deep vein thrombosis
